# Post-harvest processed parsnip showed improved anti-oxidative capacity and protective potential against acrolein-induced inflammation *in vitro* and *in vivo*

**DOI:** 10.3389/fnut.2024.1507886

**Published:** 2024-11-20

**Authors:** Kangwook Lee, Jeong Hoon Pan, La Yoon Choi, Jaehyun Ju, Brandy Le, Liana C. Williams, Tae Jin Cho, Eunjin Lee, Ji Soo Yoon, Chae Lee Park, Sang-Yoon Kim, Sung Hum Yeon, Jeonghoon Kim, Mulim Choi, Kongsik Kim, Kee-Hong Kim, Jae Kyeom Kim

**Affiliations:** ^1^Department of Food and Biotechnology, Korea University, Sejong, Republic of Korea; ^2^Department of Food Science and Nutrition, and The Basic Science Institute of Chosun University, Chosun University, Gwangju, Republic of Korea; ^3^Department of Food Science, Purdue University, West Lafayette, IN, United States; ^4^Department of Health Behavior and Nutrition Sciences, University of Delaware, Newark, DE, United States; ^5^R&D Center, Huons Co. Ltd., Ansan, Republic of Korea; ^6^EFIL BioScience Inc., Seongnam, Republic of Korea

**Keywords:** acrolein, parsnip, post-harvest processes, respiratory inflammation, antioxidant activity

## Abstract

**Introduction:**

Post-harvest processing plays a crucial role in enhancing the bioactive properties of vegetables. This study aimed to investigate the impact of post-harvest aging on parsnip’s bioactive profile and its protective effects against acrolein (Acr)-induced inflammation, a common pollutant and irritant linked to respiratory inflammation.

**Methods:**

Parsnips (*Pastinaca sativa* L.) were aged at 60°C for up to 30 days, with extracts collected at intervals. Total phenolic content (TPC) and antioxidant capacity were assessed using DPPH assays. Key bioactive compounds, including falcarindiol, DDMP, and 5-HMF, were quantified. In vitro studies used BEAS-2B cells to evaluate anti-inflammatory effects, while *in vivo* tests involved treating Acr-exposed mice with aged parsnip extract to observe cytokine responses.

**Results:**

Aged parsnip extracts showed a 9.96-fold increase in TPC and a 4.25-fold increase in antioxidant capacity after 30 days. Bioactive compounds significantly increased in aged samples, especially falcarindiol and 5-HMF. *In vitro*, aged parsnip reduced Acr-induced TNF-α and IL-1β expression. *In vivo*, treated mice showed reduced bronchial inflammation, goblet cell hyperplasia, and cytokine expression compared to controls.

**Discussion:**

These findings suggest that post-harvest aging enhances parsnip’s antioxidant and anti-inflammatory properties, highlighting its potential as a functional food ingredient for managing inflammation and respiratory health.

## Introduction

Post-harvest processes play a crucial role in determining the quality, shelf-life, and nutritional value of crops and other edible or medicinal vegetables. These processes include a series of treatments such as cleaning, curing, drying, and storage that are employed to maintain the post-harvest integrity of the vegetables. The primary objective of these treatments is to minimize physiological degradation, microbial contamination, and biochemical changes that can adversely affect the vegetables’ nutritional and medicinal properties. The effectiveness of post-harvest treatments is influenced by several factors, including the type of vegetable, the specific conditions of the treatment process, and the subsequent storage environment; as an example, controlled atmospheres (e.g., low oxygen levels and high carbon dioxide concentrations) are critical for maintaining the quality and extending the shelf-life of vegetables [e.g., broccoli (1)].

Root crops, such as carrots, ginseng, and parsnips, also undergo significant biochemical changes during post-harvest handling. For instance, drying, which reduces the water activity of vegetables, is a critical post-harvest process that inhibits the growth of spoilage microorganisms and preserves the nutritional quality of the produce [reviewed in (2)]. Similarly, post-harvest processes for nutraceutical vegetables, such as garlic and ginseng, are equally essential to preserve their therapeutic properties. For instance, black garlic is produced by subjecting fresh garlic bulbs to prolonged heating under controlled humidity. This process not only enhances the antioxidant properties of garlic but also alters its chemical composition, resulting in increased levels of certain bioactive compounds such as S-allylcysteine (3). Moreover, Red ginseng is produced through steaming and drying of fresh ginseng, a process that enhances its pharmacological properties. This treatment induces substantial alterations in the ginsenoside profile, resulting in improved therapeutic efficacy compared to unprocessed ginseng.

Parsnips (*Pastinaca sativa* L.) are root vegetables belonging to the Apiaceae family, together with carrots and celeries. This biennial plant is cultivated primarily for its large, fleshy taproot, which has been utilized for both culinary and medicinal purposes since ancient times. Parsnips are appreciated for their sweet and nutty flavor, making them a popular ingredient in various culinary applications. Nutritionally, parsnips are rich in vitamins as well as dietary fibers, which contribute to immune support, bone health, and overall cellular function (4). In addition to their nutrient density, parsnip contain diverse bioactive compounds contributing to their health-promoting properties. These include polyacetylenes like falcarinol and falcarindiol, which exhibit anti-inflammatory and anti-cancer activities [reviewed in (5)]. Parsnips also contain furanocoumarins, which, despite being phototoxic in large quantities, may offer medicinal benefits when consumed moderately, particularly in treating skin conditions and enhancing immune responses (reviewed in (5)). We recently demonstrated that raw parsnip and celery supplementation can protect mice from acrolein-induced pulmonary injury (6). However, despite extensive investigation into parsnips and their bioactive compounds, knowledge gaps persist regarding the impact of post-harvest processing on (1) quantities of phytonutrients and (2) preventive potential against inflammatory responses.

## Materials and methods

### Post-harvest process of parsnip and preparation of extract samples

**Figure 1:**
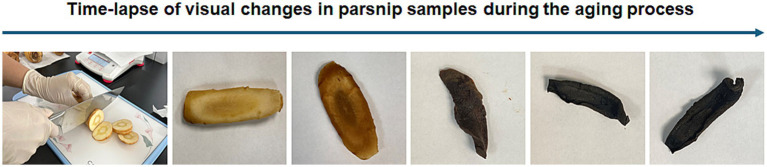
Changes in parsnip appearance over time due to post-harvested processing.

Parsnips were purchased from a local store in Wilmington, DE, USA. Once obtained, the outer skin of the parsnips is lightly removed, and the washed parsnips were cut into coins approximately 0.5 cm thick. After parsnips were placed in a convection oven, which was set at 60°C, up to 30 days (Figure 1). The samples were retrieved at Day 10, Day 20, and Day 30 and then subjected to extraction. Specifically, the aged parsnips were immersed in 30% ethanol in water at a 1:5 ratio and then extraction was carried out at room temperature (20–25°C) in a shaking incubator (125 rpm) for 24 h. The supernatant was filtered (Whatman No. 4; Sigma-Aldrich, St. Louis, MO). The remaining precipitated parsnips were extracted again under the same conditions and filtered, then the extracts were combined. The combined extract was concentrated under reduced pressure at 35°C using a rotary evaporator and finally purged with nitrogen to produce the sample.

### Total phenolic contents of aged parsnip

The parsnip extracts prepared above were prepared at concentrations of 2.5, 5.0, and 10.0 mg/ml and then each sample (100 μl) was mixed with 200 μl of 2% Na_2_CO_3_ and reacted for 3 min, followed by the addition of 10 μl of 50% Folin–Ciocalteu reagent and a further 3-min reaction. The absorbance was measured at 720 nm. The sample solvent (15% DMSO) mixed with 2% Na2CO3 for 3 min served as the blank, and the mixture with the 50% Folin reagent for an additional 3 min served as the control. The TPC was calculated and compared against a standard curve prepared with gallic acid [also known as gallic acid equivalent (GAE); Supplementary Figure S1].

### 2,2-Diphenyl-1-picrylhydrazyl radical scavenging activity of aged parsnip

Using the parsnip extracts prepared above, samples were prepared at concentrations of 2.5, 5.0, and 10.0 mg/ml. After, each sample (20 μl) was mixed with 80 μl of 0.3 mM DPPH reagent (Sigma-Aldrich) and allowed to react in the dark at room temperature for 1 h. The absorbance was measured at 517 nm. The sample solvent (10% DMSO) mixed with 100% ethanol for 1 h served as the blank, and the sample solvent mixed with 0.3 mM DPPH reagent for 1 h served as the control. The radical scavenging activity was calculated using the following formula:


$$\begin{array}{l} \mathrm{Radical}\;\mathrm{scavenging}\;\mathrm{activity}\;\left(\%\right)=\\ \frac{\mathrm{Absorbance}\;\mathrm{of}\;\mathrm{control}-\mathrm{Absorbance}\;\mathrm{of}\;\mathrm{sample}}{\mathrm{Absorbance}\;\mathrm{of}\;\mathrm{control}\text{.}} \end{array}$$

### Monitoring changes of bioactives in aged parsnip samples

In order to monitor changes in active compounds during the post-harvest process, we have assessed a few known representative constituents present in apiaceous vegetables. First, a major furanocoumarin, 8-methoxypsolaren (8-MOP) was quantified. To construct a standard curve, 8-MOP standard (Sigma-Aldrich) was dissolved it in 1 ml of methanol and then filtered through a 0.2 μm Whatman PVDF syringe filter. To prepare parsnip samples, 30% ethanolic extracts were dissolved in methanol to achieve a concentration of 10 mg/ml. To make sure samples were completely dissolved in the solvent (i.e., methanol), the solution was placed in an ultrasonic bath for 1 min and then filtered through the same syringe filter. The instrumental analysis for 8-MOP was conducted using a ZORBAX Eclipse Plus C18 column (5 μm, 4.6 mm × 250 mm; Agilent technology, Santa Clara, CA) maintained at 30°C. The PDA detector set to 320 nm was used, with a sample injection volume of 10 μl. The mobile phase consisted of solvent A (water) and solvent B (methanol), delivered at a flow rate of 1 ml/min. The gradient elution program was as follows: 60% A/40% B initially and at 5 min, 40% A/60% B at 15 min, 20% A/80% B at 25 min, 15% A/85% B at 30 min, returning to 60% A/40% B at 31 min and held until 35 min.

Next, a major polyacetylene, falcarindiol present in parsnip extract, was quantified. For the standard curve, falcarindiol standard (Chemfaces; Wuhan, China) was dissolved in methanol and then filtered using a 0.2 μm Whatman PVDF syringe filter. For falcarindiol quantification, Ultrapure water was added to the parsnip extracts to achieve a concentration of 50 mg/ml. After, liquid–liquid extraction was performed with an equal volume of ethyl acetate, and the ethyl acetate layer was collected; this liquid–liquid extraction was repeated three times and then the fractions were pooled followed by nitrogen evaporation. The concentrated extract was dissolved in methanol to achieve a concentration of 100 mg/ml, and the solution was filtered through the same syringe filter. The instrumental analysis was performed using a CAPCELL PAK C18 UG120 column (4.6 × 250 mm, 5 μm; Osaka Soda, Osaka, Japan) maintained at 30°C, with a PDA detector set to 210 nm and an injection volume of 10 μl. The mobile phase consisted of solvent A (water) and solvent B (acetonitrile), delivered at a flow rate of 1 ml/min. The gradient elution program was as follows: 80% A/20% B initially and at 5 min, 50% A/50% B at 10 min, 47% A/53% B at 30 min, 35% A/65% B at 45 min, 25% A/75% B at 70 min, maintained at 25% A/75% B until 72 min, 5% A/95% B at 90 min, held until 95 min, 0% A/100% B at 100 min, returning to 80% A/20% B at 110 min.

2,3-dihydro-3,5-dihydroxy-6-methyl-4H-pyran-4-one (DDMP) and 5-hydroxymethyl furfural (5-HMF) were quantified. For quantification, DDMP and 5-HMF standards were purchased from the Chemspace (South Brunswick, NJ). The instrumental analysis was performed using an Agilent 1,260 Infinity system equipped with a UV detector set at 280 nm. An Agilent Eclipse C18 column (250 mm × 4.6 mm, 5 μm; Agilent Technologies) was used, maintained at a temperature of 30°C. The mobile phase consisted of solvent A (0.1% trifluoroacetic acid in water) and solvent B (methanol), delivered at a flow rate of 1 ml/min with an injection volume of 10 μl. The gradient elution program was as follows: 95% A/5% B initially, changed to 60% A/40% B at 30 min, then to 0% A/100% B at 35 min, and maintained at 0% A/100% B until 40 min.

### BEAS2 cell culture

The BEAS-2B cells were chosen because they exhibit characteristics of human bronchial epithelial cells, making them suitable for studying respiratory inflammation models. The cells were obtained from the American Type Culture Collection (ATCC, Manassas, VA) and maintained in a culture medium composed of a 1:1 mixture of LHC-9 and RPMI 1640, supplemented with 10% fetal bovine serum, 1% penicillin/streptomycin, and additional factors such as 25 ng/ml of hEGF. The cells were cultured at 37°C in a humidified incubator with 5% CO_2_. Regular monitoring was conducted to check for cell confluency, morphological integrity, and contamination. When cells reached 80–90% confluency, they were sub-cultured using 0.25% trypsin–EDTA solution. Cell pellets were then resuspended in fresh medium and transferred to new culture plates. Medium renewal was performed every 2 to 3 days to ensure optimal growth conditions.

### Cell viability assessment via 3-(4,5-dimethylthiazol-2-yl)-2,5-diphenyltetrazolium bromide (MTT) assay

Cell viability was assessed using the MTT assay (Sigma-Aldrich) after treating BEAS-2B cells with different concentrations of extracted samples. The goal of this method was to optimize activity while minimizing toxicity. Cells were plated at a density of 50,000 cells per well and allowed to grow for 24 h. After, the variously diluted samples were treated (i.e., up to 10 mg/ml concentration), and the cells were incubated for another 24 h. Following this, the culture medium was removed, and the cells were washed with phosphate-buffered saline. Each well was then treated with 100 μl of MTT solution (0.5 mg/ml) in complete medium for 2 h. After incubation, the MTT formazan crystals were dissolved with 100 μl of DMSO, and the absorbance was measured at 570 nm.

### Anti-inflammatory potential of aged parsnip extract in BEAS-2B human bronchial epithelial cells

Based on the MTT assay result, the BEAS-2B cells were treated with aged parsnip (2.5 mg/ml) for 24 h followed by treatment with acrolein (Acr; 160 μM) for additional 24 h to induce inflammatory responses. After, the cells were harvested and then subjected to total RNA extraction.

### Animal study design, diet preparation, and Acr treatment

For the animal intervention study, a total of 20 male C57BL/6 J mice were assigned to either a negative control [NEG group; American Institute of Nutrition (AIN)-93G], positive control (POS group; AIN-93G + Acr) or AP intervention group (AP 20D group; 20 day-aged parsnip extract intervention + AIN-93G + Acr). The AIN-93G diet was used as a basal diet for all groups (7). For the AP group, the animals were administered with aged parsnip extract via oral gavage once daily over 2 weeks at a dosage of 10 mg of extract per kg of body weight. The mice used in this study were 5-week-old male C57BL/6J mice. Male mice were selected due to their heightened airway inflammatory and functional responses to LPS compared to female mice (8). To make the study’s findings relevant to humans, we calculated the human equivalent dose. For a 70 kg adult, the equivalent dose would be the consumption of about 10 g of fresh parsnip per day, considering an extraction yield of approximately 7%. This calculation helps demonstrate how the doses used in the study could be applicable to human dietary habits. This dosage is within the range of typical dietary intake, making it clinically relevant and feasible for human applications. The dosage aligns with common dietary intake, making it feasible and clinically applicable. Additionally, from day 9 through day 14, Acr was administered at a dose of 10 μmol/kg body weight for five consecutive days to induce inflammatory effects. During the treatment period, assigned diets remained the same. For Acr treatment, following isofluorane exposure for 5–10 s until unconscious, Acr was administered intranasally, as we described previously (6). For Acr preparation, Acr was dissolved in saline, and administration volume did not exceed 15 μl. The NEG group mice were exposed to the same isofluorane condition followed by Acr-free normal saline solution. After, all mice were euthanized by exsanguination, and harvested tissues were weighed and stored in RNALater (Thermo Fisher Scientific, Waltham, MA) at −80°C or fixed in 10% neutral buffered formalin solution before further analyses. All animal handling and experiments were performed following protocols approved by the Institutional Animal Care and Use Committee of the University of Delaware (Protocol Approval No. 1359-2022-A).

### Assessments of pulmonary pathology

Lung tissues were harvested, and then fixed in 10% neutral buffered formalin for 24 h and subsequently dehydrated using ethanol and xylene. After embedding in paraffin, the specimens were cut into 5 μm sections with a microtome. Hematoxylin and eosin (H&E) staining was applied to the lung tissue to examine epithelial thickness. Furthermore, periodic acid-Schiff (PAS) staining (Abcam, Cambridge, MA) was performed to detect mucin in goblet cells, facilitating the visualization of goblet cell hyperplasia.

### Quantitative PCR

Using the RNeasy Plus kit (Qiagen, Hilden, Germany), total RNA was extracted from the collected BEAS-2B cells and lung tissues. Subsequently, cDNA was synthesized employing the PrimeScript™ RT reagent Kit (Takara Bio; Shiga, Japan). Each cDNA sample underwent analysis via TaqMan real-time quantitative PCR. The PCR reactions were conducted utilizing TaqMan™ Fast Advanced Master Mix (Applied Biosystems; Waltham, MA) along with pre-designed TaqMan primers for genes of interest. All steps involving RNA extraction, cDNA synthesis, and quantitative PCR were carried out following the manufacturer’s protocols.

### Assessment of blood inflammation markers

The Proteome Profiler Mouse Cytokine Array Panel A (ARY006; R&D Systems, Minneapolis, MN) was employed to measure serum inflammatory cytokine profiles; procedures were performed according to the manufacturer’s instructions.

### Statistical analysis

Statistical evaluations were conducted using GraphPad Prism software (Boston, MA, USA). We assessed whether the data followed a normal distribution using the Shapiro–Wilk test. For data that did not follow a normal distribution, we applied the Kruskal-Wallis test followed by Dunn’s multiple comparisons test. Conversely, for data that conformed to a normal distribution, we used a one-way ANOVA followed by Dunnett’s post-hoc test. We presented the data as mean ± standard deviation (SD) or standard error of means (SEM) where appropriate. The results were considered statistically significant if *p* values were less than 0.05.

## Results

### Post-harvest process of parsnip improved antioxidative potential *in vitro*

**Figure 2:**
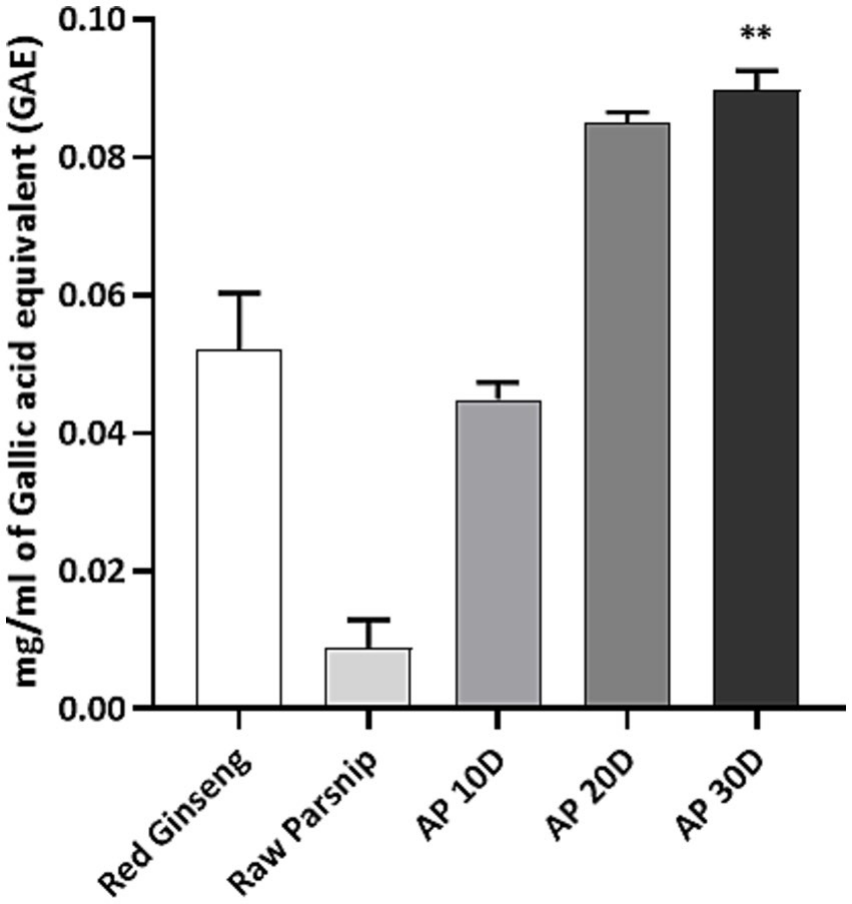
Comparing the total phenolic content (TPC; mg gallic acid equivalent/ml) of red ginseng, raw parsnip and aged parsnips. Normality was assessed using the Shapiro–Wilk test, and the data did not follow a normal distribution. Therefore, significance was assessed using the Kruskal-Wallis test followed by Dunn’s multiple comparisons test. Results are shown as means ± standard deviations (*n* = 3). **p < 0.01 (vs. Raw Parsnip). AP 10D, Aged parsnip over 10 days; AP 20D, Aged parsnip over 20 days; AP 30D, Aged parsnip over 30 days; GAE, Gallic acid equivalent; TPC, Total phenolic content.

We investigated the impact of post-harvest processing on the phenolic content and antioxidant capacity of parsnip. As reference controls, raw parsnip and red ginseng extract groups were compared (Figure 2). Overall, the results demonstrated a significant enhancement in TPC of parsnips, subjected to the post-harvest process, with a clear trend showing increased levels of phenolics over time. Specifically, the TPC, expressed as mg TPC/ml of GAE, was assessed for red ginseng, raw parsnip, and parsnip aged for 10, 20, and 30 days (namely, AP 10D, AP 20D, and AP 30D in Figure 2). The results were as follows: red ginseng exhibited a phenolic content of 0.0522 ± 0.008 mg/ml GAE, raw parsnip showed 0.0089 ± 0.004 mg/ml GAE, AP 10D had 0.0449 ± 0.003 mg/ml GAE, AP 20D demonstrated 0.0849 ± 0.002 mg/ml GAE, and AP 30D revealed the highest phenolic content at 0.0897 ± 0.003 mg/ml GAE. The statistical significance was notable, compared to the raw parsnip, AP 30D showed significantly higher phenolic content with *p* < 0.01 (Figure 2). While the AP 20D sample also showed an increased mean phenolic content compared to the raw parsnip, the observed difference (*p* = 0.0548) was close to what is often considered a meaningful change. These results demonstrate a significant increase in phenolic content as the aging period extended, with AP 30D showing the highest content, nearly double that of red ginseng and more than four times that of raw parsnip.

**Figure 3:**
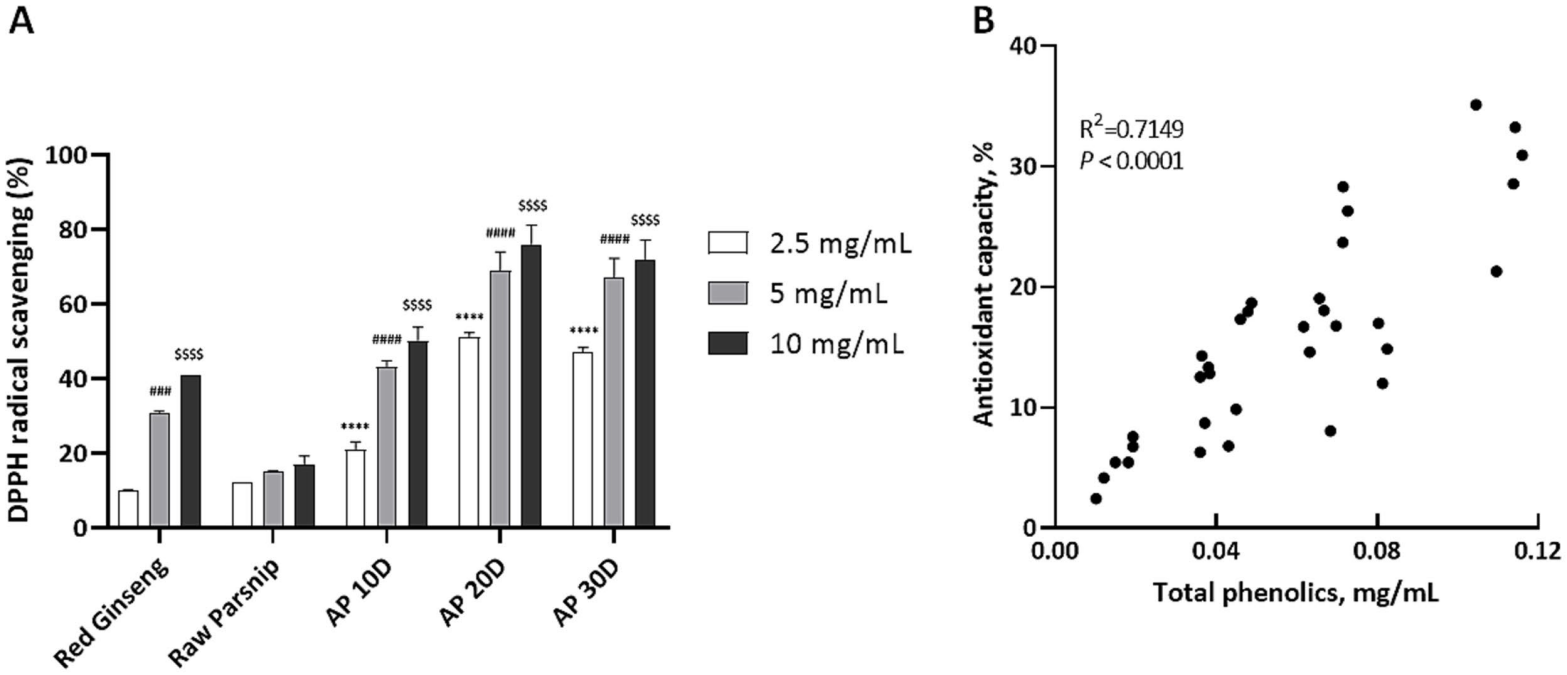
Comparative analysis of antioxidant activity and phenolic content in red ginseng, raw parsnip, and aged parsnips: 2,2-diphenyl-1-picrylhydrazyl (DPPH; %) and Pearson Correlation. (A) Comparing DPPH (%) of red ginseng, raw parsnip, and aged parsnips (*n* = 3). Normality was assessed using the Shapiro–Wilk test, and the data followed a normal distribution. Therefore, significance was assessed using one-way ANOVA followed by Dunnett’s *post-hoc* test. Results are shown as means ± standard deviations (*n* = 3). ****p < 0.0001 (vs. 2.5 mg/ml of Raw Parsnip); ###*p* < 0.001, ####*p* < 0.0001 (vs. 5 mg/ml of Raw Parsnip); $$$$*p* < 0.0001 (vs. 10 mg/ml of Raw Parsnip). (B) The scatter plot illustrates the Pearson correlation between TPC (*x*-axis) and DPPH radical scavenging activity (*y*-axis) of the samples. Each point represents an individual sample. The Pearson correlation coefficient (r = 0.7149) indicates a strong positive linear relationship between TPC and DPPH values, suggesting that higher TPC is associated with higher DPPH activity. The line of best fit is included to visually demonstrate this correlation. AP 10D, Aged parsnip over 10 days; AP 20D, Aged parsnip over 20 days; AP 30D, Aged parsnip over 30 days; GAE, Gallic acid equivalent; TPC, Total phenolic content.

We also investigated the impact of post-harvest processing on parsnip’s antioxidant capacity using the DPPH radical scavenging activity assay (Figure 3A). The results indicated a significant enhancement in antioxidant capacity as the post-harvest aging period extended. DPPH radical scavenging activity was measured at three concentrations (2.5, 5, and 10 mg/ml) for red ginseng, raw parsnip, and aged parsnip samples (AP 10D, AP 20D, and AP 30D).

Red ginseng displayed antioxidant capacities of approximately 10, 31, and 41% at 2.5, 5, and 10 mg/ml, respectively. Raw parsnip showed lower capacities of about 12, 15, and 17% at the same concentrations. Aging parsnips for 10 days (AP 10D) increased DPPH radical scavenging capacities to roughly 21, 43, and 50% at 2.5, 5, and 10 mg/ml, respectively.

Further aging for 20 days (AP 20D) resulted in even higher antioxidant capacities of approximately 51, 69, and 76% at 2.5, 5, and 10 mg/ml, respectively. However, these capacities did not increase proportionally in the AP 30D samples, which presented about 47, 67, and 72% DPPH radical scavenging activities at 2.5, 5, and 10 mg/ml concentrations, respectively. Based on these results, we adopted the AP 20D sample for *in vitro* and *in vivo* applications.

The enhancement in antioxidant capacity observed with aging can be attributed to the increased concentration of phenolic compounds. In order to test this hypothesis, we performed the Pearson correlation analysis between TPC contents and DPPH radical scavenging activities. As results, the two variables were highly correlated with the R^2^ value of 0.7149 and *p* < 0.0001 (Figure 3B).

### Characterization of potential bioactives in aged parsnip extract

In order to explore specific changes in bioactives in parsnip extract, we have assessed the contents of a few potential constituents and their changes over the period of aging process. First, 8-MOP was quantified which is one of representative furanocoumarins present in apiaceous vegetables (5). Interestingly, we noted that raw parsnip extract contained higher 8-MOP than aged parsnip extract (Table 1). Second, per our analytical chemistry analysis, the content of falcarindiol was dramatically increased (0.005 vs. 0.141 mg/g; Table 1) in aged parsnip extract, which may partially explain the enhanced *in vitro* bioactivity results. To the best of my knowledge, this is the first report demonstrating that post-harvest processes enhanced the contents of polyacetylenes in parsnip. Lastly, since we have subjected heat to the parsnip roots, we speculated if such conditions increased secondary metabolites of Maillard reaction. Two major Maillard reaction intermediate compounds were quantified: 5-HFM and DDMP (9). We found that aged parsnip extract contains much higher 5-HMF content than raw parsnip (2.10 vs. 0.17 mg/g; Table 1). DDMP content was 2.04 mg/g raw parsnip yet it was increased to 3.16 mg/g aged parsnip which is consistent with improved DPPH radical scavenging activity result. These findings suggest that post-harvest aging significantly alters the bioactive profile of parsnips, potentially enhancing their antioxidant properties.

**Table 1:** Comparison of the bioactive contents in raw and aged parsnips.

Sample name	8-MOP (mg/g)	5-HMF (mg/g)	DDMP (mg/g)
Raw parsnip^1^	0.183 ± 0.016	0.170 ± 0.000	2.040 ± 0.000
Aged parsnip^2^	0.070 ± 0.001	2.100 ± 0.000	3.160 ± 0.000

Data are expressed as mean ± standard deviation (*n* = 3). DDMP, 2,3-dihydro-3,5-dihydroxy-6-methyl-4H-pyran-4-one; 5-HMF, 5-hydroxymethyl furfural; 8-MOP, 8-methoxypsolaren.

1 Raw parsnip, unprocessed parsnip.

2 Aged parsnip, parsnip processed as described in the Materials and Methods section.

### A protective function of aged parsnip extract against Acr-induced inflammation *in vitro*

**Figure 4:**
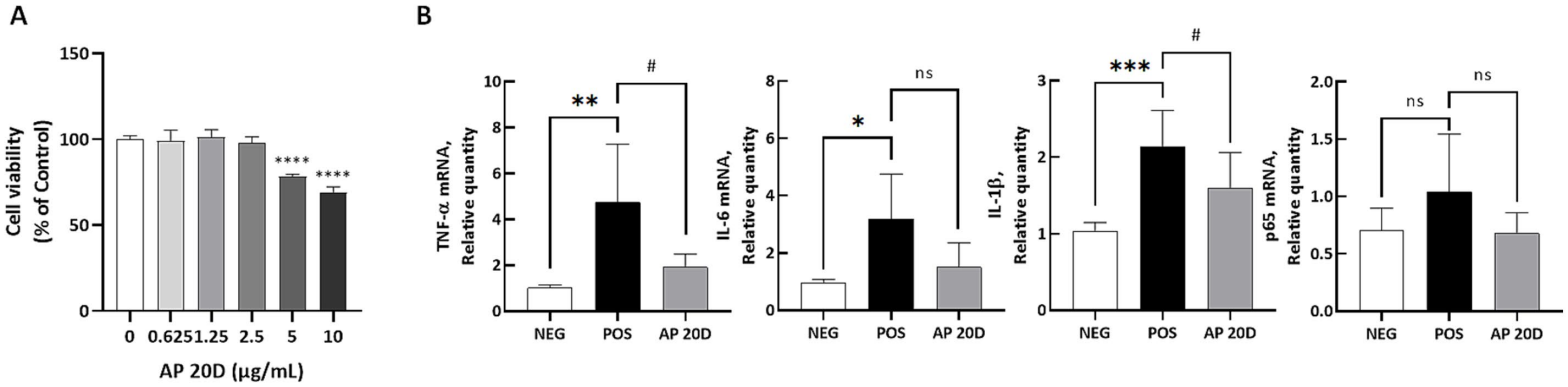
Dose-dependent effects of AP 20D on cell viability and inflammatory marker expression in human bronchial/tracheal epithelial cells. (A) Dose-dependent effects of AP 20D on the viability of human primary bronchial/tracheal epithelial cells. Normality was assessed using the Shapiro–Wilk test, and the data followed a normal distribution. Therefore, significance was assessed using one-way ANOVA followed by Dunnett’s post-hoc test. Results are shown as means ± standard deviations (*n* = 3). ****p < 0.0001 (vs. 0). The “0” group refers to the “Control” group. (B) Quantitative PCR results for TNF-α, IL-6, IL-1β, p65, and p53 in human bronchial epithelial cells. Normality was assessed using the Shapiro–Wilk test. For data that followed a normal distribution, significance was assessed using one-way ANOVA followed by Dunnett’s post-hoc test. For data that did not follow a normal distribution, the Kruskal-Wallis test followed by Dunn’s multiple comparisons test was applied. Results are shown as means ± standard deviations (*n* = 6). *p < 0.05, **p < 0.01, ***p < 0.001 (vs. NEG); #*p* < 0.05 (vs. POS). ‘ns’ indicates statistically not significant. NEG, negative group; POS, acrolein-treated group; AP 20D, POS group treated with 20-day-aged parsnip.

Acr (2-propenal) is a pervasive pollutant generated from the incomplete combustion of organic matter (10) and is one of the most abundant and reactive aldehydes in cigarette smoke (11). Acr primarily targets tissues at the site of contact, such as the respiratory tract during inhalation (12). As a reactive aldehyde, Acr generates reactive oxygen species, leading to oxidative stress (13). This oxidative stress triggers several critical consequences, including increased epithelial permeability, lipid peroxidation, and depletion of the antioxidant glutathione in the alveoli, which can lead to inflammation and tissue injury (14). Evidence suggests that Acr exposure elevates the risk of cancers as well as inflammatory diseases (15). To test the role of aged parsnip in Acr-induced inflammation, we first examined the cytotoxicity of aged parsnip (i.e., AP 20D, as justified above) in BEAS-2B cells. As shown in Figure 4A, the aged parsnip showed no toxicity in BEAS-2B cells up to 2.5 mg/ml concentration while significant cytotoxicity was observed at 5 mg/ml or higher.

Next, BEAS-2B cells were treated with the AP 20D sample at 2.5 mg/ml concentration for 24 h followed by treatment with Acr (160 μM) for additional 24 h to induce inflammatory responses. A few representative mRNA markers related to the NF-κB signaling (Figure 4B). Specifically, *TNF-α*, *IL-6*, *IL-1β*, and *p65* were quantified where *TNF-α*, *IL-6*, and *IL-1β*, were all increased in response to the Acr treatment (i.e., POS in the Figure 4B) compared to the NEG. This indicates that Acr exposure induces a strong inflammatory response, consistent with its known role in causing oxidative stress and inflammation. Importantly, the AP 20D group showed a significant reduction in *TNF-α* expression compared to the POS group (*p* < 0.01), suggesting that the aged parsnip extract effectively mitigates Acr-induced inflammation. Similar patterns were reproduced for *IL-1β* (*p* < 0.05); *IL-1β* is a crucial mediator of inflammatory responses, and its reduction highlights the prevention potential of AP in combating Acr-induced inflammation (14) On the other hand, there was no significant difference in *IL-6*, and *p65* between POS and AP 20D groups.

### Aged parsnip extract was protective against Acr-induced inflammation *in vivo*

**Figure 5:**
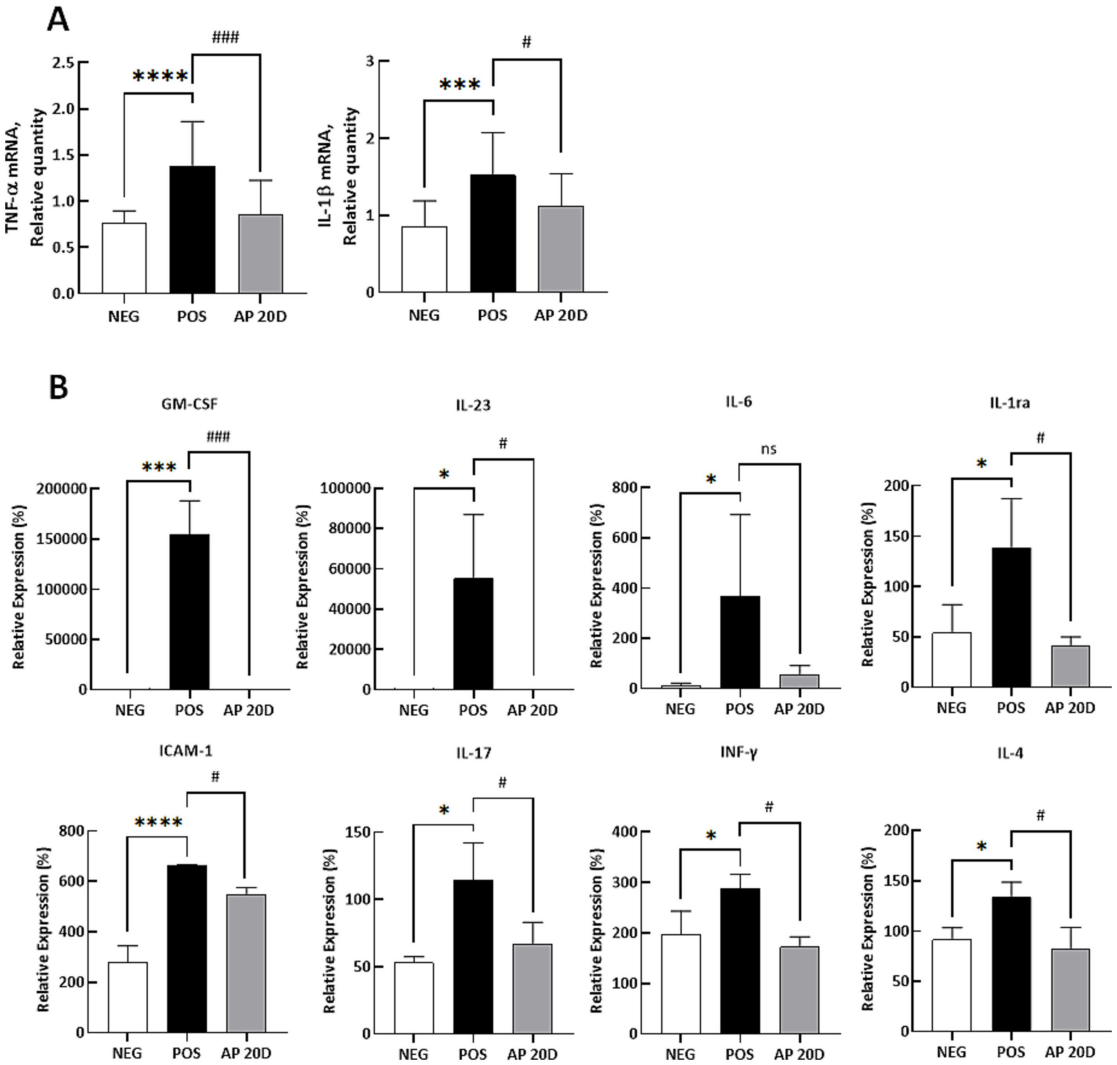
Quantitative PCR and ELISA results for inflammatory markers in mouse lung tissue and blood samples. (A) Quantitative PCR results for TNF-α and IL-1β in mouse lung tissue. Normality was assessed using the Shapiro–Wilk test, and all data followed a normal distribution. Therefore, significance was assessed using one-way ANOVA followed by Dunnett’s post-hoc test. Results are shown as means ± standard deviations (*n* = 6–8). ***p < 0.001, ****p < 0.0001 (vs. NEG), #*p* < 0.05, ###*p* < 0.001 (vs. POS). (B) ELISA results for GM-CSF, IL-23, IL-6, IL-1RA, ICAM-1, IL-17, IFN-γ, and IL-4 in mouse blood. Normality was assessed using the Shapiro–Wilk test, and all data followed a normal distribution. Therefore, significance was assessed using one-way ANOVA followed by Dunnett’s post-hoc test. Results are shown as means ± standard deviations (*n* = 3–4). *p < 0.05, ***p < 0.001, ****p < 0.0001 (vs. NEG), #*p* < 0.05, ###*p* < 0.001 (vs. POS). ‘ns’ indicates statistically not significant. NEG, negative group; POS, acrolein-treated group; AP 20D, POS group treated with 20-day-aged parsnip.

Based on the *in vitro* results, we further examined the effects of AP 20D on inflammatory responses after intranasal treatment of Acr in C57BL/6J mice. Specifically, the AP 20D was orally administered every day for 2 weeks at the dose of 10 mg extract/kg body weight. Given that our extraction process for aged parsnip achieved a yield of approximately 7% based on fresh parsnip weight, the dosage we provided to the mice (i.e., 10 mg extract/kg body weight) translates to an equivalent dosage for an adult weighing 70 kg as the consumption of about 10 grams of fresh parsnip (16). With this exposure level, we demonstrated that both *TNF-α* and *IL-1β* mRNA expressions were decreased in the AP 20D intervention group, compared to the POS (*p* < 0.001 and *p* < 0.05, respectively; Figure 5A). Additionally, we further explored cytokine proteins expression in serum samples; the Figure 5B shows the relative protein expression levels of various cytokines and adhesion molecules, including GM-CSF, IL-23, IL-6, IL-1ra, ICAM-1, IL-17, INF-*γ*, and IL-4, in the serum samples of mice subjected to Acr exposure and subsequent treatment with the AP 20D. Briefly, GM-CSF, IL-23, IL-6, IL-1ra, ICAM-1, IL-17, INF-γ, and IL-4 protein expression levels were significantly increased in the POS group compared to the NEG group (GM-CSF: *p* < 0.001; IL-23: *p* < 0.05; IL-6: *p* < 0.05; IL-1ra: *p* < 0.05; ICAM-1: *p* < 0.0001; IL-17: *p* < 0.05; INF-γ: *p* < 0.05; and IL-4: *p* < 0.05). In contrast, treatment with aged parsnip extract (i.e., AP 20D) significantly reduced the levels of these markers compared to the POS group, except for IL-6, which did not show a statistical significance. The results demonstrate that Acr exposure significantly upregulates the expression of several pro-inflammatory cytokines and adhesion molecules in mouse; such inflammatory responses, induced by the Acr, are consistent with previous studies showing that Acr induces oxidative stress and inflammation, leading to elevated cytokine production (11). In contrast, the administration of AP 20D significantly reduced the levels of these inflammatory markers in Acr-exposed mice, which align well with our *in vitro* findings, where AP treatment significantly reduced Acr-induced expression of TNF-*α* and IL-1β in human bronchial epithelial cells. Both sets of data suggest that AP 20D effectively attenuates inflammation induced by Acr, not only by reducing the expression of TNF-α and IL-1β but also by downregulating a broad range of other pro-inflammatory cytokines and adhesion molecules.

**Figure 6:**
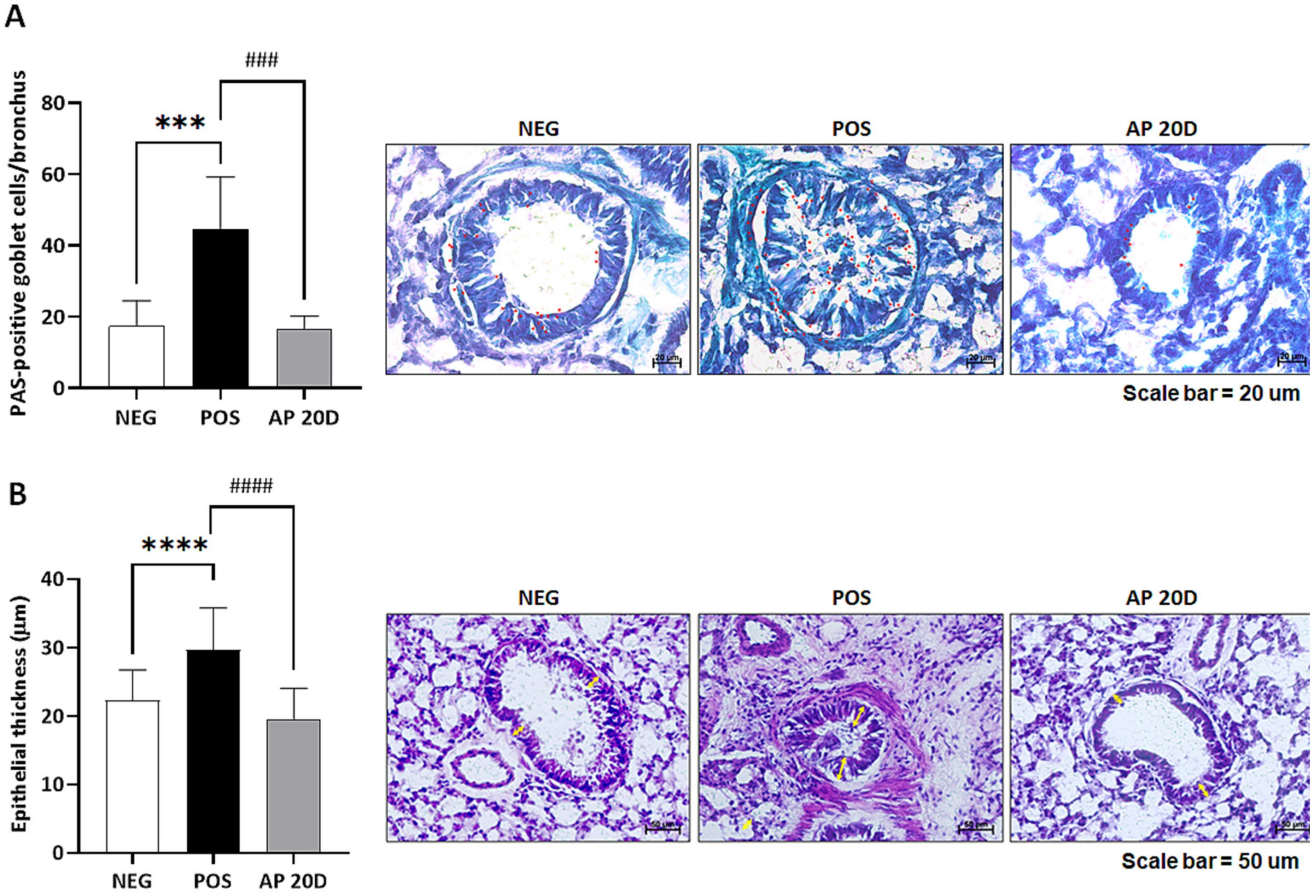
Representative lung histology sections stained with (A) Periodic Acid-Schiff (PAS) and (B) Hematoxylin and eosin (H&E). Red-headed arrow points to magenta-colored mucus-positive staining (scale bar indicates 20 μm). Yellow double-headed arrow denotes bronchi thickness (scale bar represents 50 μm). Normality was assessed using the Shapiro–Wilk test, and all data followed a normal distribution. Therefore, significance was assessed using one-way ANOVA followed by Dunnett’s post-hoc test. Results are shown as means ± standard deviations (*n* = 3–4). ***p < 0.001, ****p < 0.0001 (vs. NEG), ###*p* < 0.01, ####*p* < 0.0001 (vs. POS). NEG, negative group; POS; acrolein-treated group; AP 20D, POS group-treated with 20-day-aged parsnip.

We also examined pathophysiological changes in lung tissues in mice. First, the PAS shows the effects of Acr exposure and AP 20D intervention on mucin-secreting goblet cells in the bronchial epithelium of mice. The quantitative analysis of PAS-positive goblet cells per bronchus reveals that the number of PAS-positive goblet cells was significantly higher in the POS group compared to the NEG group (*p* < 0.001; Figure 6A). In contrast, the administration of AP 20D reversed the number of PAS-positive goblet cells (*p* < 0.001). The images show representative sections stained for mucin, with more intense staining and higher goblet cell counts in the POS group, indicating increased mucin production. The AP 20D group showed reduced staining and goblet cell counts, similar to the NEG group. The PAS staining results illustrate the impact of Acr exposure on mucin secretion by goblet cells in the bronchial epithelium and the mitigating effects of aged parsnip extract (i.e., AP 20D). The significant increase in PAS-positive goblet cells in the POS group indicates that Acr exposure induces hyperplasia of goblet cells and excessive mucin production, which is consistent with the known effects of Acr, as a potent respiratory irritant (11). However, the PAS-positive goblet cells in the lung tissues were reduced in the AP 20D group; this aligns with our earlier findings where AP 20D administration reduced the expression of systemic pro-inflammatory cytokines (TNF-α and IL-1β) *in vivo*, indicating its broad anti-inflammatory and protective properties. The results suggests that administration of aged parsnip may help maintain airway integrity and function by mitigating the pathological changes induced by Acr exposure.

Lastly, our H&E staining results, along with the quantitative analysis of epithelial thickness, demonstrate the impact of Acr exposure and AP 20D administration on bronchial epithelial structure in mice lung tissues. In brief, the POS group exhibited significantly increased epithelial thickness compared to the NEG group (*p* < 0.0001) which such abnormal changes were reversed in the AP 20D group (*p* < 0.0001; Figure 6B). This thickening of the bronchial walls is indicative of inflammatory responses and airway remodeling, which are common features of chronic respiratory diseases such as asthma and chronic obstructive pulmonary disease (COPD); the Acr is known to cause oxidative stress and inflammation, leading to structural changes in the respiratory tract (15). The significant reduction in epithelial thickness observed in the AP 20D-treated group is consistent with earlier *in vitro* and other *in vivo* markers (e.g., the expression of pro-inflammatory cytokines and mucin-secreting goblet cells). The ability of aged parsnip to reduce bronchial wall thickening indicates its potential to prevent or ameliorate the pathological remodeling of airway structures induced by Acr exposure (17). The thickening of bronchial walls in the POS group is a result of increased cellular proliferation and inflammatory cell infiltration, responses to the oxidative stress and damage caused by Acr (13). The protective effects of AP 20D in our mice study may be attributed to its antioxidant properties, which help neutralize reactive oxygen species and reduce oxidative stress, preventing structural changes associated with chronic inflammation.

## Discussion

Our post-harvest aging process significantly boosts the antioxidant capacity of parsnip; the clear trend observed across different concentrations further reinforces the importance of optimizing post-harvest processing techniques to maximize the nutritional benefits of parsnip. For comparison, Golubkina et al. (18) examined the biochemical characteristics of processed parsnip roots versus garlic bulbs under high temperature and humidity (18). Their findings indicated that the TPC was increased by approximately 6.3 folds while DPPH radical scavenging activity was enhanced by 2.8 folds. In our study, the TPC and DPPH radical scavenging activity were increased up to 9.96 and 4.25 folds, respectively (for AP 30D sample). This increase in TPC is likely attributed to enzymatic reactions during aging, as biosynthesis typically ceases post-harvest. Although there is a difference in values between the studies, our findings are consistent with the trends observed by Golubkina et al.; our study employed aging at drying heat (i.e., 60°C) for 10, 20, and 30 days, whereas Golubkina et al. used a more intense process with conditions of 70°C and 95% relative humidity for 4 weeks which might be limited to industrial applications.

Our results monitoring changes of bioactives in aged parsnip samples showed that post-harvest aging led to a decrease in 8-MOP content while significantly increasing the levels of bioactive compounds such as falcarindiol, 5-HMF, and DDMP. Interestingly, we noted that raw parsnip extract contained higher 8-MOP than aged parsnip extract (Table 1). Although it is unclear how the content of this furanocoumarin was decreased, it is possible to postulate that improved antioxidative activities of aged parsnip was not contributed by the 8-MOP. Nevertheless, future studies warrant the impact of post-harvest aging to the content of furanocoumarins in parsnip. Second, polyacetylenes are a class of organic compounds characterized by multiple carbon–carbon triple bonds within their molecular structure. These compounds are found in various plants and are known for their bioactive properties, including anti-inflammatory, antimicrobial, and anticancer activities [reviewed in (19)]. In particular, polyacetylenes such as falcarinol and falcarindiol have been studied for their potential health benefits and are notable for their presence in apiaceous vegetables including parsnips (19). Lastly, the Maillard reaction intermediate compound 5-HMF, formed from the dehydration of hexoses like glucose and fructose under thermal conditions, serves as an indicator of heat processing and has been studied for its potential antioxidant properties (9). 5-HMF is a key intermediate compound formed during the Maillard reaction and is produced from the dehydration of hexoses like glucose and fructose, under thermal conditions (20). This compound serves as an indicator of heat processing (21) and has been studies for its potential antioxidant properties (22). In our study, the increased 5-HMF content in aged parsnip suggests that heat treatment plays a crucial role in enhancing its bioactive profile. Similarly, the formation of DDMP specifically involves the enolization and subsequent dehydration of glucose or fructose in the presence of amino acids. This reaction occurs prominently under low moisture conditions, which favor the 2,3-enolization pathway over the 1,2-enolization pathway, thus promoting the formation of DDMP (23). In the context of food processing and particularly in the processing of vegetables like parsnips, DDMP can be detected when these foods are subjected to conditions that promote the Maillard reaction, such as high temperature over an extended period (23, 24).

Based on the *in vivo* results, our study demonstrates that aged parsnip extract (AP 20D) effectively mitigates Acr-induced inflammation and oxidative stress in a mouse model, further supporting its potential as a therapeutic agent. Specifically, the AP 20D treatment significantly reduced the mRNA expression levels of pro-inflammatory cytokines such as TNF-α and IL-1β in lung tissues, aligning with previous research highlighting the role of these cytokines in Acr-induced inflammatory responses (14). Acr is known to generate reactive oxygen species (ROS), leading to oxidative stress and subsequent inflammation (13), which results in increased cytokine production, lipid peroxidation, and damage to the respiratory epithelium (15). In our study, the reduction of these markers upon treatment with AP 20D suggests that its antioxidant properties effectively neutralize ROS and reduce oxidative stress, preventing the cascade of inflammatory responses typically triggered by Acr exposure (11). Furthermore, the AP 20D treatment led to a decrease in goblet cell hyperplasia and bronchial epithelial thickness, which are hallmark signs of airway remodeling often associated with chronic respiratory diseases like asthma and COPD (17). These findings suggest that aged parsnip extract’s ability to modulate both cellular and structural responses to Acr exposure may play a crucial role in preserving lung tissue integrity and function, providing a promising approach to managing inflammation and oxidative stress in the respiratory tract.

The observed anti-inflammatory and antioxidative effects of aged parsnip extract (AP 20D) in both cell and animal models can be attributed to the synergistic actions of its bioactive compounds, particularly falcarindiol, 5-HMF, and DDMP. Falcarindiol, a known polyacetylene, has been shown to suppress the activation of the NF-κB signaling pathway, which is a crucial regulator of pro-inflammatory cytokines such as TNF-α and IL-1β (5). This inhibition likely contributes to the reduced expression of these cytokines in both BEAS-2B cells and mouse lung tissues treated with AP 20D. Additionally, 5-HMF, a Maillard reaction product, plays a significant role in mitigating oxidative stress by scavenging ROS, thereby preventing ROS-mediated damage to epithelial cells and reducing oxidative stress markers *in vivo* (9). These combined actions not only attenuate the inflammatory response but also protect the respiratory epithelium from structural damage, as evidenced by the reduced goblet cell hyperplasia and bronchial epithelial thickening observed in acrolein-exposed mice. Moreover, DDMP, another Maillard reaction product, further enhances the antioxidative properties of the extract by contributing to the neutralization of ROS (23). Together, these bioactive compounds provide a multifaceted mechanism that underlies the protective effects of aged parsnip extract against acrolein-induced oxidative stress and inflammation.

This study presents several strengths, particularly in demonstrating the beneficial effects of post-harvest processed parsnip extract on mitigating inflammation and oxidative stress induced by Acr exposure; one major strength is the comprehensive approach that combines *in vitro* and *in vivo* experiments, which provides robust evidence supporting the protective effects of the aged parsnip against Acr-induced respiratory inflammation and tissue remodeling. The use of multiple markers for inflammation, oxidative stress, and tissue pathology further supports our findings. Another strength is the detailed analysis of bioactive compounds in aged parsnip extract, which helps elucidate the potential mechanisms underlying the observed protective effects; as aforementioned, to the best of our knowledge this is the first report to quantify several bioactives over the process. However, the study also has some limitations. One key limitation is the lack of specific identification and quantification of all possible bioactive compounds that might contribute to the observed effects. While the study focuses on a few known compounds like 8-MOP, falcarindiol, DDMP, and 5-HMF, other potentially important bioactives may have been overlooked. Moreover, the relatively short duration of the *in vivo* experiments limits our understanding of the long-term effects of aged parsnip extract. Future studies should investigate whether the observed protective effects are sustained over longer periods and if prolonged exposure leads to any adverse effects. Additionally, while the study demonstrates the effects of aged parsnip extracts in a mouse model, further research might be warranted to confirm these findings in humans and to evaluate the safety and efficacy in clinical settings.

In conclusion, the present study highlights the significant potential of post-harvest processed parsnip extract in mitigating Acr-induced respiratory inflammation and oxidative stress. The enhanced phenolic content and antioxidant capacity of aged parsnip extracts correlate with their ability to reduce pro-inflammatory cytokines and prevent bronchial wall thickening in both *in vitro* and *in vivo* models. Further research is necessary to fully understand the mechanisms of action, optimize dosing, and evaluate the clinical relevance of these findings.

## Data Availability

The original contributions presented in the study are included in the article/Supplementary material, further inquiries can be directed to the corresponding authors.
