# Supplementary figures and images for: Post-harvest processed parsnip showed improved anti-oxidative capacity and protective potential against acrolein-induced inflammation in vitro and in vivo
Source: Front Nutr. 2024 Nov 20;11:1507886. doi: 10.3389/fnut.2024.1507886 (PMC11614627; doi:10.3389/fnut.2024.1507886)

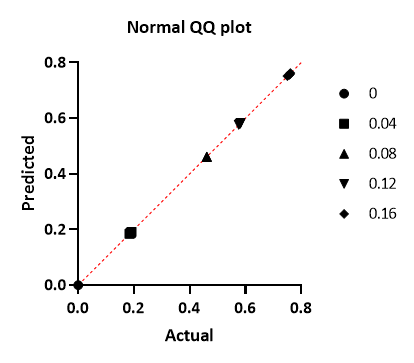

Supplement: Supplementary file 1 [file Image_1.TIF]
